# Chitosan/siRNA nanoparticle targeting demonstrates a requirement for single-minded during larval and pupal olfactory system development of the vector mosquito *Aedes aegypti*

**DOI:** 10.1186/1471-213X-14-9

**Published:** 2014-02-19

**Authors:** Keshava Mysore, Emily Andrews, Ping Li, Molly Duman-Scheel

**Affiliations:** 1 Department of Medical and Molecular Genetics, Indiana University School of Medicine, South Bend, IN 46617, United States of America; 2 Eck Institute for Global Health, University of Notre Dame, Notre Dame, IN 46556, USA; 3 Department of Biological Sciences, University of Notre Dame, Notre Dame, IN 46556, USA

**Keywords:** *Aedes aegypti*, Neural development, Single-minded, Odorant receptor, Olfactory receptor neuron, Targeting, siRNA, Nanoparticle, Mosquito, Olfaction

## Abstract

**Background:**

Essentially nothing is known about the genetic regulation of olfactory system development in vector mosquitoes, which use olfactory cues to detect blood meal hosts. Studies in *Drosophila melanogaster* have identified a regulatory matrix of transcription factors that controls pupal/adult *odorant receptor (OR)* gene expression in olfactory receptor neurons (ORNs). However, it is unclear if transcription factors that function in the *D. melanogaster* regulatory matrix are required for *OR* expression in mosquitoes. Furthermore, the regulation of *OR* expression during development of the larval olfactory system, which is far less complex than that of pupae/adults, is not well understood in any insect, including *D. melanogaster*. Here, we examine the regulation of *OR* expression in the developing larval olfactory system of *Aedes aegypti*, the dengue vector mosquito.

**Results:**

*A. aegypti* bears orthologs of eight transcription factors that regulate *OR* expression in *D. melanogaster* pupae/adults. These transcription factors are expressed in *A. aegypti* larval antennal sensory neurons, and consensus binding sites for these transcription factors reside in the 5’ flanking regions of *A. aegypti OR* genes. Consensus binding sites for Single-minded (Sim) are located adjacent to over half the *A. aegypti OR* genes, suggesting that this transcription factor functions as a major regulator of mosquito *OR* expression. To functionally test this hypothesis, chitosan/siRNA nanoparticles were used to target *sim* during larval olfactory development. These experiments demonstrated that Sim positively regulates expression of a large subset of *OR* genes, including *orco*, the obligate co-receptor in the assembly and function of heteromeric OR/Orco complexes. Decreased innervation of the antennal lobe was also noted in *sim* knockdown larvae. These *OR* expression and antennal lobe defects correlated with a larval odorant tracking behavioral defect. *OR* expression and antennal lobe defects were also observed in *sim* knockdown pupae.

**Conclusions:**

The results of this investigation indicate that Sim has multiple functions during larval and pupal olfactory system development in *A. aegypti*.

## Background

The genetics of olfactory system development is largely unexplored in most non-model insect species, including hematophagous disease vector mosquitoes, which use olfactory cues to detect blood meal hosts. To address this issue, we have begun a large-scale effort to develop the dengue and yellow fever vector mosquito *Aedes aegypti* as a model for studying vector mosquito neurodevelopmental biology [1]. Our recent study demonstrated that chitosan/siRNA targeting can be used to knockdown genes during mosquito larval and pupal development [2]. Here, this methodology is applied to assess how odorant receptor (OR) expression is regulated in olfactory receptor neurons (ORNs) during *A. aegypti* larval development.

The coordinated developmental regulation of ORN targeting and OR expression, both of which are critical to the sense of smell, dictates what odors will be detected by a neuron and which behaviors are elicited in response to these odors [3]. Research in the genetic model insect *Drosophila melanogaster* has provided insight into how these two processes are regulated during pupal development [4-6]. *D. melanogaster* ORNs are located in the antenna and maxillary palp. These ORNs typically express one of 60 possible *OR* genes, the choice of which is determined through a process that produces a stereotyped receptor to neuron map [3]. Systematic and genetic analysis of the regulation of *Drosophila OR* expression in pupae and adults has suggested that each *OR* gene has a “zip code” which consists of enhancer elements that act positively to promote expression of particular *ORs* in some neurons, as well as elements that restrict *OR* expression in others [7]. Recent work, including a large-scale RNAi-screen, has revealed a number of transcription factors that bind these regulatory elements to regulate *OR* gene expression in *Drosophila*[6,8,9]. These cis-regulatory factors are differentially expressed in ORNs and required for proper regulation of the expression of *OR* genes [6,7]. The particular combination and levels of expression of these cis-regulators of transcription in specific neurons generates the *OR* regulatory matrix, a code governing which particular *OR* gene is expressed and which are repressed in any given ORN. Ultimately, ORNs expressing the same *OR* gene project axons that converge on the same glomerulus, one of several spheroidal modules located in the antennal lobe of the insect brain [10,11].

The insect larval olfactory system mimics the architecture of the olfactory system found in pupae and adults, but is reduced in cell number and therefore less complex [12,13]. This reduced complexity makes the larval antennal lobe an excellent tissue in which to track olfactory system development. It is presently unclear if any of the transcription factors that function to regulate *OR* expression in *D. melanogaster* pupae/adults are required for *OR* expression in larvae. Moreover, although there is evidence that *Drosophila* larval ORNs expressing the same *OR* project to similar areas of the larval brain [14], it is unclear how this process is regulated, or if a regulatory matrix exists for this less sophisticated larval olfactory system. Our recent study detailed ORN targeting in the developing *A. aegypti* larval olfactory system [2]. Here, the regulation of *OR* gene expression is examined in the developing *A. aegypti* larval antenna.

This investigation focuses on functional characterization of the *A. aegypti* ortholog of the transcription factor Single-minded (Sim). Although Sim is known to regulate *OR* expression in *Drosophila* pupae/adults [6], its function has not been assessed in the developing larval olfactory system. Moreover, a requirement for Sim to regulate *OR* gene expression has not yet been assessed during olfactory development in other insects, including mosquitoes. Furthermore, a requirement for Sim in the regulation of ORN innervation of the antennal lobe has not yet been described in any insect species, including *Drosophila*. Here, we use chitosan/siRNA-mediated knockdown to test the hypothesis that Sim is required for olfactory system development in *A. aegypti* larvae and pupae*.* The results of this study suggest that Sim function is required for proper *OR* expression and antennal lobe development during both the larval and pupal stages of *A. aegypti* development.

## Results

### Expression and chitosan/siRNA nanoparticle-mediated knockdown of *sim* during *A. aegypti* olfactory development

**Figure 1:**
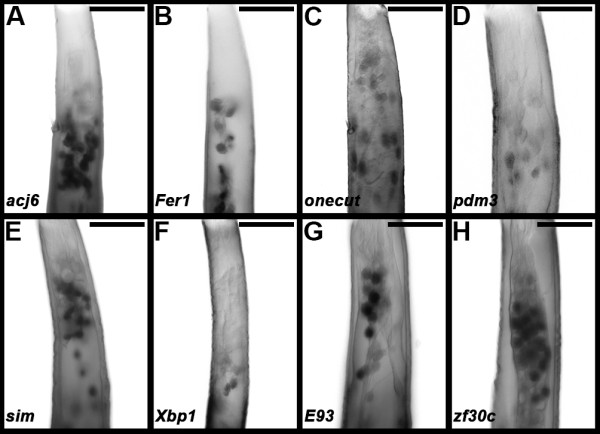
**Transcription factor expression in the *A. aegypti *larval antenna.** Expression of the eight indicated transcription factors is detected in *A. aeygpti* fourth instar larval antennae **(A-H)**. Each transcription factor is expressed in a subset of *A. aegypti* larval antennal sensory neurons, and expression levels of the transcription factors vary from neuron to neuron within this subset. The proximal ends of antennae are oriented upward in all panels. Scale bars = 25 microns.

A search for consensus binding sites in the 5’ flanking sequences of the 115 *A. aegypti OR* genes [15] uncovered multiple consensus binding sites for the transcription factors Acj6, Fer1, Onecut, Pdm3, Xbp1, and Sim (Table 1). These data suggested that the transcription factors function to regulate *A. aegypti OR* gene expression. In support of this notion, expression of each transcription factor was detected in developing antennal ORNs (Figure 1). Furthermore, expression of *Aae E93 (AAEL004572)* and *Aae zf30C (AAEL004774), Aedes* orthologs of two transcription factors that regulate *Drosophila OR* gene expression [6], was also detected in developing antennae (Figure 1; consensus binding sites for the protein products of these transcription-factor encoding genes have not yet been reported). Expression of each of the eight transcription factors is detected in a subset of *A. aegypti* antennal ORNs, and expression levels of each gene vary from neuron to neuron within this subset. These expression data (Figure 1), in conjunction with detection of consensus binding sites adjacent to *Aedes OR* genes (Table 1), suggest that the transcription factors may be required for *A. aegypti OR* expression.

**Table 1:** **Consensus binding sites for transcription factors in 5’ flanking regions of *A. aegypti OR *** **genes**

**TF**	***Aae *** **gene ID**	**Class**	**Consensus binding site**	**# flanking sites**
**Acj6**	*AAEL005507*	POU-homeobox	GACTTGAATAAATTAAAACTTT	55
**Fer1**	*AAEL008660*	bHLH	CACCTG(N)TTTCCCA	44
**Onecut**	*AAEL002359*	Cut-Homeobox	WTATTGATTW	22
**Pdm3**	*AAEL004094*	POU	TAATGA	49
**Sim**	*AAEL011013*	PAS-bHLH	CACGT	64
**Xbp1**	*AAEL005558*	bZIP	TCACGT	22

Consensus binding site sequences [6,19-24] for six transcription factors (TFs) expressed in the developing *A. aegypti* antenna are noted. Numerous consensus binding sites (# Flanking Sites) for these transcription factors were found in the 1 kB 5’ flanking sequences upstream of the open reading frame (ORF) of the 115 annotated *A. aegypti OR* genes [15]. Of these, Sim was observed to have at least one binding site in over half of the *OR* genes, suggesting it might be one of the major regulators of *OR* transcription in *A. aegypti.*

**Figure 2:**
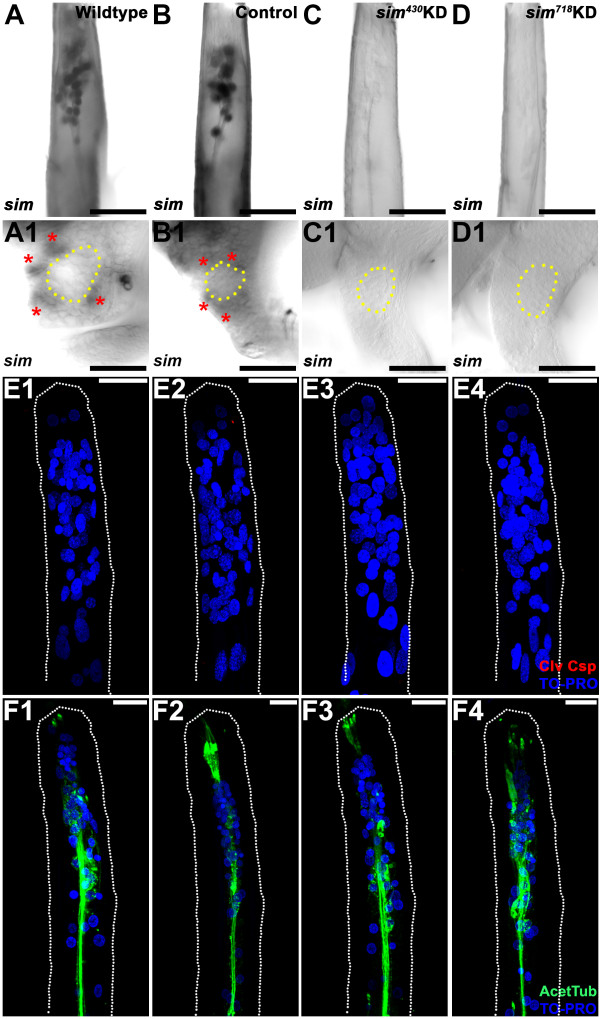
**Expression and siRNA-nanoparticle mediated knockdown of *sim *in the larval olfactory system.***sim* is expressed in the antennae **(A)** and brain **(A1)** of wildtype L4 larvae. Two *sim* siRNAs, *sim*^*430*^**(C, C1)** and *sim*^*718*^**(D, D1)**, were found to effectively knockdown (KD) *sim* in both L4 antennae **(C, D)** and brains **(C1, D1)** when delivered to *A. aegypti* larvae via chitosan nanoparticle feeding. Control siRNA feedings did not impact *sim* expression (antenna in **B**; brain in **B1**). In wildtype **(A1)** and control-fed animal **(B1)** L4 brains, clusters of cells expressing *sim* transcripts (red asterisks) are detected adjacent to the antennal lobe, the boundaries of which are marked by the yellow dotted circles in panels **A1**-**D1**. The overall reduced number of antennal sensory neurons targeting to the antennal lobes in *sim* knockdown animals does not appear to result from cell death, as evidenced by the lack of cleaved-caspase 3 staining in the L4 antennae of *sim* knockdown animals (**E3**,**4**; compare to wildtype and control-fed animals in **E1**,**2**; nuclei are marked by TO-PRO in **E**). Note that while no cleaved-caspase 3 was detected in the antennae shown, positively labeled cells were detected in other wild-type *A. aegypti* pupal tissues (not shown), suggesting that the reagent employed in these studies is effective in *A. aegypti.* Acetylated tublin staining (green, F) revealed normal antennal sensory neuron axon bundles in *sim*^*430*^ and *sim*^*718*^ knockdown animals (compare to wildtype and control-fed antennae in **F1** and **F2**, respectively). The proximal ends of antennae are oriented upward in panels **A**-**D**, **E**, and **F**. Dorsal is oriented upward in panels **A1**-**D1**. Scale bars = 25 microns.

Sim consensus binding sites were identified in the 5’ flanking sequences of approximately half of the *OR* genes (Table 1). Further analysis of *sim* expression in the developing olfactory system indicated that in addition to being expressed in L4 antennal sensory neurons (Figures 1E and 2A), *sim* is transcribed in the L4 brain, where it is expressed in clusters of cells adjacent to the larval antennal lobe (Figure 2A1). Based on these observations, we hypothesized that Sim is required for *A. aegypti* olfactory system development and pursued siRNA-mediated targeting of *Aae sim* to test the hypothesis.

siRNAs *sim*^*430*^ or *sim*^*718*^ were delivered to larvae via chitosan/nanoparticles mixed with their food with the goal of knocking down *sim* expression in the developing olfactory system. siRNA nanoparticles containing a sequence lacking significant homology to any *A. aegypti* gene served as a control in all experiments. Control siRNA feedings did not have a noticeable impact on *sim* expression (Figure 2B,B1). *In situ* hybridization demonstrated that siRNA-mediated knockdown of *sim* was attained in both the brain and antenna when larvae fed on nanoparticles containing either siRNAs *sim*^*430*^ (Figure 2C,C1) or *sim*^*718*^ (Figure 2D,D1). qRT-PCR assays with pooled brains dissected from whole animals indicated that in comparison to brains from control-nanoparticle fed animals, *sim* knockdown brains had on average a 47% reduction in *sim* transcripts (p = 0.005; n = 6). Knockdown levels observed in the antennae, in which a 77% reduction in *sim* levels was observed with respect to control-fed animals (p = 0.002; n = 3), were even higher. Despite some variability in the levels of knockdown between tissues and between animals, which is typical in RNAi experiments, *in situ* hybridization experiments indicated that *sim* transcripts were undetectable in half of the knockdown animals following treatment with *sim*^*430*^ or *sim*^*718*^ siRNA (Figure 2C,C1,D,D1; information concerning n numbers for this study and all knockdown phenotypes described below are reported in the Methods section). Thus, use of the chitosan/siRNA knockdown technique permitted knockdown of *Aae sim* and analysis of its function during olfactory system development. Moreover, use of the two separate *sim* knockdown siRNAs throughout the investigation helped to ensure that the phenotypes generated were not simply the result of off-site targeting by either siRNA.

### Sim is required for *OR* gene expression in the developing *A. aegypti* larval antenna

**Figure 3:**
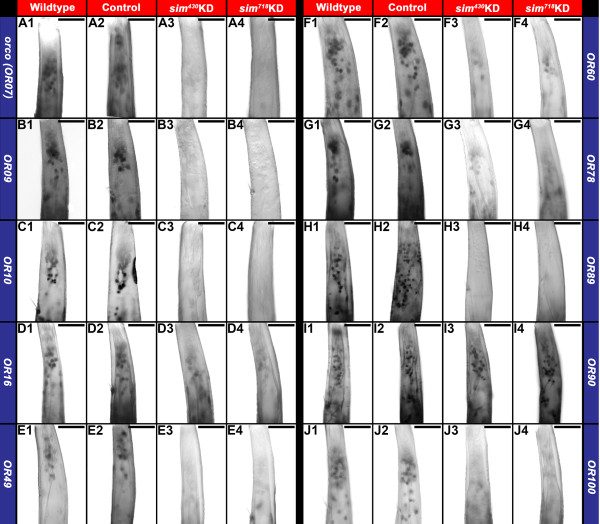
***sim *is required for *OR *gene expression in the *A. aegypti *larval antenna.***orco* and *ORs 9, 10, 16, 49, 60, 78, 89, 90,* and *100* transcripts are detected in wildtype (**A1**-**J1**, respectively) and control-fed (**A2**-**J2**, respectively) L4 antennae. Downregulated expression of *orco* and *ORs 9, 10 16, 49, 60, 78, 89,* and *100*, all of which have adjacent Sim consensus binding sites, is observed in animals that were fed with chitosan/siRNA *sim*^*430*^ (**A3**-**H3**, **J3** respectively) or *sim*^*718*^ (**A4**-**H4**, **J4**, respectively). *orco***(A3, A4)**, *OR9***(B3,B4)**, *OR10***(C3, C4)**, *OR49***(E3,E4)**, and *OR89***(H3, H4)** expression is not detectable in *sim* knockdown individuals, while expression of *ORs 16***(D3, D4)**, *60***(F3, F4)**, *78***(G3, G4)**, and *100***(J3, J4)** is reduced in comparison to wildtype **(A1-H1, J1)** and control-fed **(A2-H2, J2)** larvae. Expression of *OR90***(I1-I4)**, which lacks adjacent Sim consensus binding sites, is unchanged in knockdown animals **(I3, I4)**. The proximal ends of antennae are oriented upward in all panels. Scale bars = 25 microns.

The reduced complexity of the mosquito larval olfactory system, which mimics the adult architecture [2], makes it an excellent tissue in which to assess the impacts of *sim* knockdown on *OR* expression. For example, only a subset of the 115 *A. aegypti OR* genes has been detected in the larval antenna, including 24 detected by qRT-PCR [15], and an additional four *(ORs 10, 28, 49* and *100)* which were detected here through *in situ* hybridization experiments in the L4 antenna (Figure 3). Consensus binding sites for Sim were identified in 11 of the 28 *OR* genes expressed in the larval antenna (Table 2), including *orco* (formerly *OR7*) and *ORs 9, 10, 16, 28, 49, 60, 78, 89, 92a,* and *100*. Expression of these ORs was assessed through *in situ* hybridization in fourth instar *sim* knockdown larval antennae. These experiments revealed that although expression of these genes could be detected in wildtype and control-fed L4 animals, transcripts of *orco*, *OR9*, *OR10*, *OR49*, and *OR89* (Figure 3A,B,C,E,H, respectively) could not be detected in *sim* knockdown L4 animals, while expression of *ORs 16*, *60*, *78*, and *100* (Figure 3D,F,G,J) is reduced in comparison to wildtype and control-fed larvae. In contrast, expression of *OR90* (Figure 3I), which lacks an adjacent Sim binding site, was not impacted by knockdown of *sim*. In addition to demonstrating that loss of *OR* expression in *sim* knockdown animals correlates with the presence of Sim binding sites, this result demonstrated that *sim* knockdown antennal sensory neurons, which do express *OR90,* do not simply die in response to *sim* knockdown. This was further confirmed through nuclear staining and a lack of expression of the apoptosis marker Cleaved caspase-3 in *sim* knockdown antennae (Figure 2E3,E4). Moreover, acetylated tubulin staining of antennae revealed normal antennal sensory neuron axon bundles in *sim* knockdown antennae (Figure 2F3,F4), suggesting that axonogenesis initiates properly in *sim* knockdown ORNs. In summary, the expression of *OR* genes bearing flanking Sim consensus binding sites was compromised during L4 antennal development in *sim* knockdown animals, but loss of *sim* did not impact cell survival or the initiation of axonogenesis in L4 ORNs.

**Table 2:** **Sim binding sites in *A. aegypti *** **larval *OR *** **genes**

***Aae *** **gene ID**	**VectorBase gene name**	**Sim consensus sites in 1kB 5’ flanking sequence**	**Sim consensus sites in first intron**
*AAEL016970*	*GPROR1*	-	-
*AAEL005999*	*GPROR2*	-	-
*AAEL017138*	*GPROR3*	-	-
*AAEL005776*	*GPROR7 (orco)*	1	1
*AAEL012254*	*GPROR8*	-	-
*AAEL006005*	*GPROR9*	1	-
*AAEL006003*	*GPROR10*	1	-
*AAEL008442*	*GPROR14*	-	-
*AAEL008448*	*GPROR15*	-	-
*AAEL007110*	*GPROR16*	1	1
*AAEL000391*	*GPROR28*	1	-
*AAEL003395*	*GPROR34*	-	-
*AAEL003369*	*GPROR37*	-	-
*AAEL005767*	*GPROR40*	-	-
*AAEL017079*	*GPROR47*	-	-
*AAEL011895*	*GPROR48*	-	-
*AAEL001303*	*GPROR49*	1	-
*AAEL006202*	*GPROR58*	-	-
*AAEL017041*	*GPROR60*	2	1
*AAEL017277*	*GPROR61*	-	-
*AAEL006192*	*GPROR73P*	-	-
*AAEL006195*	*GPROR74*	-	-
*AAEL013419*	*GPROR75*	-	-
*AAEL013423*	*GPROR78*	1	1
*AAEL017125*	*GPROR89*	1	-
*AAEL017037*	*GPROR90*	-	-
*AAEL001310*	*GPROR92a*	1	1
*AAEL011409*	*GPROR100*	-*	-

Of the 28 *ORs* that are expressed in larvae (per [15] and additionally *ORs 10, 28, 49* and *100*, the expression of which was detected here; see Figure 2), 10 have Sim consensus binding sites in the 1 kB flanking sequences upstream of the ORF, and five of these genes also possess an additional consensus binding site for Sim in their first introns. *OR100 was found to have one 5’ flanking Sim binding site 3 kB upstream.

### Antennal lobe phenotypes in *sim* knockdown larvae

**Figure 4:**
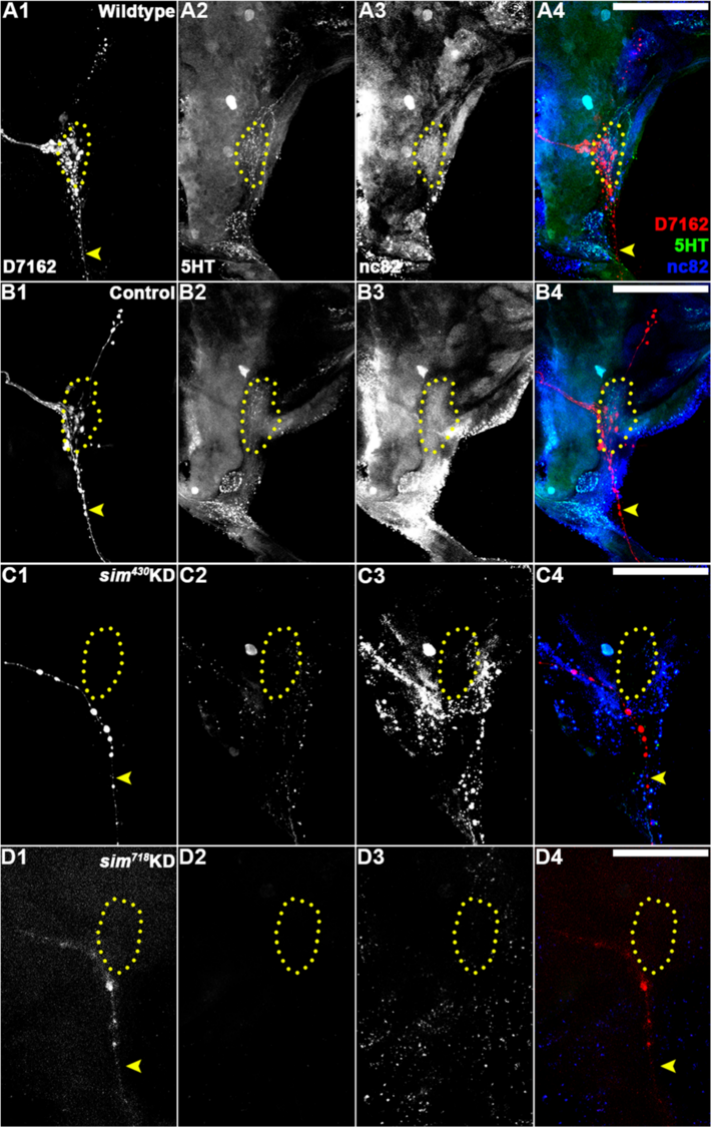
**Larval antennal lobe defects in *sim *knockdown animals.** In wildtype **(A1)** and control-fed **(B1)** L4 larvae, D7162 dye-filled antennal sensory neurons innervate the antennal lobe (highlighted by yellow dots throughout the figure), which is labeled by mAb nc82 **(A3, B3)**. Serotonergic projection neurons are labeled by anti-5HT staining in the antennal lobes of these individuals **(A2, B2)**. An overlay of the three labels is shown in panels A4 and B4 (as well as **C4**, and **D4**). *sim*^*430*^**(C1-C4)** and *sim*^*718*^**(D1-D4)** animals show a reduction in the number of antennal sensory neurons (D7162 fills in **C1**, **D1**) targeting the antennal lobe. The neuropil (nc82 label in **C3**, **D3**) and projection neurons (5HT label in **C2**, **D2**) are substantially reduced in the antennal lobes of *sim* knockdown (KD) animals. A subset of antennal sensory neurons which are believed to be gustatory receptors [2] pass through the antennal lobe and project to the subesophageal ganglion (**A1**, **B1**; yellow arrowhead). In *sim* knockdown animals (**C1**, **D1**; yellow arrowhead) these neurons are substantially reduced in number. Dorsal is oriented upward in all the panels. Scale bars = 25 microns.

The regulation of *OR* gene expression and ORN targeting are tightly coordinated during pupal olfactory system development [10,11]. It was therefore hypothesized that Sim might also function to regulate ORN targeting, even during these earlier larval stages of development. To test this hypothesis, anterograde antennal sensory neuron fills and immunohistochemical marker analyses were used to examine antennal lobe development in control-fed vs. *sim* knockdown L4 animals. As discussed previously [2], in wildtype and control-fed L4 larvae, antennal sensory neuron tracts exit the antenna (Figure 2F1,F2) and enter the developing antennal lobe region of the brain (Figure 4A1, B1). Expression of mAb nc82, which marks the synaptic neuropil in *A. aegypti*[16], labels the antennal lobe in wildtype (Figure 4A3) and control-fed (Figure 4B3) animals, where filled antennal sensory neurons synapse with projection neurons that are marked by expression of serotonin (5HT) in wildtype (Figure 4A2) and control-fed (Figure 4B2) animals. Anterograde labeling experiments detected a reduction in the number of antennal sensory neurons targeting the antennal lobe in *sim*^*430*^ (Figure 4C1) and *sim*^*718*^ (Figure 4D1) knockdown L4 animals. As discussed above, this reduced number of antennal sensory neurons in the antennal lobe does not appear to result from cell death (Figures 2E3,4 and 3I) or a lack of the initiation of axonogenesis in these neurons (Figure 2F3,F4). The *sim* knockdown L4 larvae were also assessed through staining with mAb nc82 (Figure 4C3,D3) and anti-5HT (Figure 4C2,D2) antibodies. These experiments indicated that *sim*^*430*^ (Figure 4C2,C3) and *sim*^*718*^ (Figure 4D2,D3) individuals display a decrease in overall expression of both markers within the antennal lobe region (compare to wildtype and control-fed animals in Figure 4A2,A3, 4B2,B3, respectively). These data correlated well with the reduced number of antennal sensory neurons innervating the antennal lobe in *sim* knockdown animals (Figure 4C1,D1).

### Deficient odorant tracking in *sim* knockdown animals

**Figure 5:**
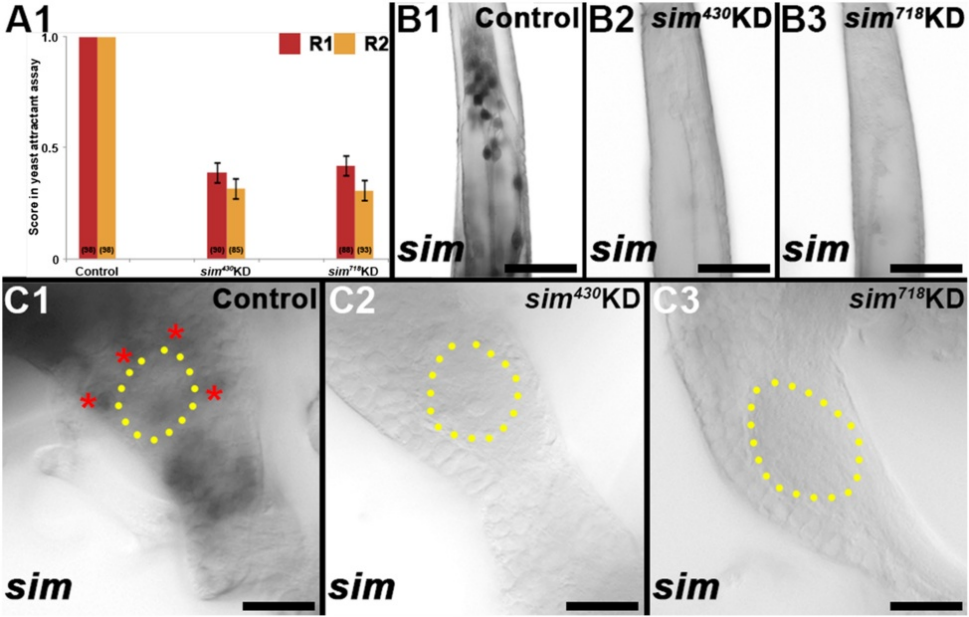
***sim *deficient larvae have a decreased yeast odorant attractant response.** Individual *sim*^*430*^ or *sim*^*718*^ knockdown (KD) vs. control-fed L4 animals were assessed for their response to a yeast odorant attractant. In each assay (replicate one = red, replicate two = orange) individual control-fed animals that were attracted to the yeast were awarded a score of 1, while animals that were not attracted to the yeast received a score of 0. Average scores for control vs. knockdown animals are plotted for each replicate experiment, for which n numbers are indicated; error bars represent standard error **(panel A1)**. The mean score for knockdown animals fed with either *sim*^*430*^ or *sim*^*718*^ was significantly lower than that of control-fed animals in both replicate experiments (p < 0.001, A1). Levels of *sim* RNA were severely reduced in the antennae **(B2, B3)** and brains **(C2, C3)** of *sim* knockdown animals (*sim*^*430*^ in B2, C2; *sim*^*718*^ in **B3**, **C3**) that failed to respond to the yeast, while normal levels of *sim* transcript were observed in control-fed antennae **(B1)** and brains (**C1**; red asterisks mark clusters of *sim* expression) of animals that were attracted to the yeast. The proximal ends of antennae are oriented upward in panels **B1**-**B3**. Dorsal is oriented upward in **C1**-**C3**, in which the antennal lobe is marked by yellow dots. Scale bars = 25 microns.

Individual *sim* knockdown and control-fed fourth instar larvae were tested in an olfactory-driven behavioral assay utilized in a recent study [2] which was modified from Liu *et al*. [17]. In the assay, control-fed and *sim* knockdown individuals were assessed for attraction to a yeast odorant pellet. All control fed animals (n = 196) touched the yeast pellet during the assay (Table 3; Figure 5A), and *in situ* hybridization experiments revealed wildtype levels of *sim* expression in the antennae and brains of these animals (Figure 5B1,C1). Knockdown of *sim* through nanoparticle delivery of either knockdown siRNA *sim*^*430*^ (p < 0.001) or *sim*^*718*^ (p < 0.001) resulted in significantly reduced performance in the yeast odorant assay (Table 3, Figure 5A). Of the individuals that fed on *sim* knockdown nanoparticles, 64% failed to touch the yeast pellet during the course of the assay (Table 3). *In situ* hybridization revealed that levels of *sim* were reduced in the antennae and brains of *sim*^*430*^ (Figure 5B2,C2) and *sim*^*718*^ (Figure 5B3,C3) individuals that failed to respond to the yeast (Table 3). *sim* transcripts could still be detected in *sim* knockdown nanoparticle-fed individuals that were attracted to the yeast (Table 3), suggesting that the *sim* knockdown levels in these animals were not sufficient enough to impact their performance in the assay. These experiments revealed that decreased levels of *sim* correlated with poor performance in a yeast odorant behavioral assay.

**Table 3:** **Levels of *sim *** **correlate with performance in a yeast behavioral assay**

		**Attracted**				**Not attracted**			
**siRNA**	**n**	**# animals**	**Normal**	**Moderate**	**Null**	**# animals**	**Normal**	**Moderate**	**Null**
**Control**	195	196 (100%)	196 (100%)	0	0	0	0	0	0
***sim***^***430***^**KD**	176	63 (35%)	48 (76%)	15 (24%)	0	113 (64%)	20 (18%)	12 (11%)	81 (71%)
***sim***^***718***^**KD**	177	66 (37%)	44 (66%)	22 (34%)	0	111 (63%)	20 (18%)	9 (8%)	82 (74%)

The data above represent a compiled summary of results obtained from four replicate experiments in which control-fed vs. *sim* knockdown (KD) animals were tested in a yeast odorant attractant behavioral assay. The total number of animals (n) indicates the number of individuals that were tested in these assays. The number of individuals (# Animals) that were attracted (left; animals that touched the yeast pellet and were awarded a score of 1) or not attracted (right; animals that did not touch the yeast pellet and received a score of 0) under each condition (Control, *sim*^*430*^ KD, or *sim*^*718*^ KD) are indicated, and the percentages of total animals are indicated following the raw numbers. *In situ* hybridization was used to assess *sim* knockdown in the brains and antennae of animals attracted (left) or not attracted (right) to the yeast. The raw number/percentage of animals with Normal (comparable to wildtype *sim* transcript levels; see Figure 5B1,C1), Null (no observable *sim* transcript; see Figure 5B2,B3,C2,C3), or Moderate (reduced but not wildtype) levels of *sim* are indicated. Loss of *sim* was found to correlate well with a lack of attraction to the yeast in this behavioral assay.

The decreased attraction of *sim* knockdown animals to the yeast pellet (Table 3, Figure 5A) correlated well with the *OR* gene expression (Figure 3) and antennal lobe (Figure 4C,D) defects noted in *sim* knockdown animals. However, given the complexity of larval feeding behavior, it is possible that the observed *sim* knockdown behavioral phenotype could result at least in part from other defects in *sim* deficient animals. Neither the control nor *sim* knockdown nanoparticle-fed animals displayed any obvious locomotor defects, suggesting that locomotor deficit was not responsible for the observed behavioral defect. It is unlikely, albeit possible, that reduced attraction could in part result from gustatory defects in *sim* knockdown larvae that might be unable to taste any trace amounts of yeast which could diffuse through the water during the course of the assay. In the L4 brain of wildtype (Figure 4A1) and control-fed (Figure 4B1) animals, a subset of antennal sensory neurons project ventrally from the antennal lobe to the subesophageal ganglion. As discussed previously [2], these neurons likely function as gustatory neurons. This subset of neurons is also substantially reduced in *sim* knockdown animals (Figure 4C1,D1). Thus, both olfactory receptor and gustatory receptor neuron defects are observed in the antennal lobes of *sim* knockdown animals (Figure 4C1,D1). These *sim* knockdown defects, in addition to *OR* gene expression defects (Figure 3), correlate well with the decreased attraction to yeast behavioral phenotype (Table 3, Figure 5A).

### *OR* expression and antennal lobe phenotypes in *sim* knockdown pupae

**Figure 6:**
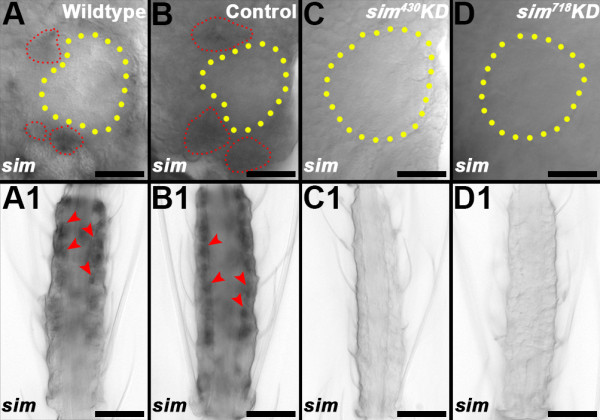
**Expression and knockdown of *sim *in pupae.***sim* is expressed in the antennal lobe **(A, B)** and antenna **(A1, B1)** of wildtype **(A, A1)** and control-fed **(B, B1)** 36 hr APF pupae. Cell clusters of *sim* expression surrounding the antennal lobe **(A, B)** are highlighted by red dots, while clusters of *sim* expression are marked by red arrowheads in the antenna **(A1, B1)**. Animals fed with knockdown (KD) siRNAs *sim*^*430*^**(C, C1)** and *sim*^*718*^**(D, D1)** lack *sim* expression in the antennal lobe region **(C, D)** and antenna **(C1, D1)**. Antennal lobes are denoted by yellow-dotted circles. Dorsal is oriented upward in panels **A**-**D**. The proximal ends of antennae are oriented upward in A1-D1. Scale bars = 25 microns.

**Figure 7:**
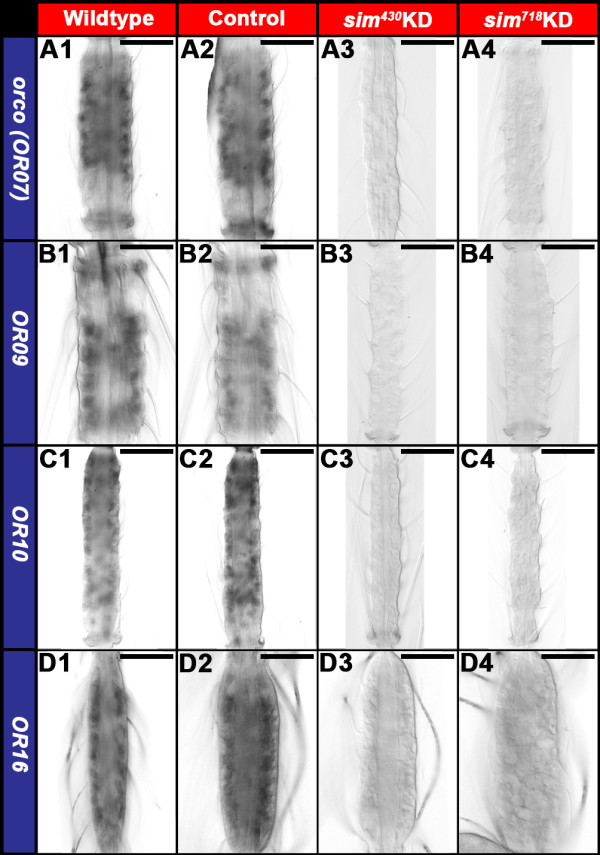
**Sim is required for *OR *gene expression in pupae.***orco* and *OR 9, 10,* and *16* transcripts are detected in wildtype (**A1**-**D1**, respectively) and control-fed (**A2**-**D2**, respectively) 36 hr APF pupal antennae. However, *orco***(A3, A4)**, *OR9***(B3,B4)**, *OR10***(C3, C4)**, and *OR16***(D3,D4)** expression is not detectable in *sim*^*430*^**(A3-D3)** or *sim*^*718*^**(A4-D4)** knockdown 36 hr APF pupal antennae. The proximal ends of antennae are oriented upward in all panels. Scale bars = 25 microns.

**Figure 8:**
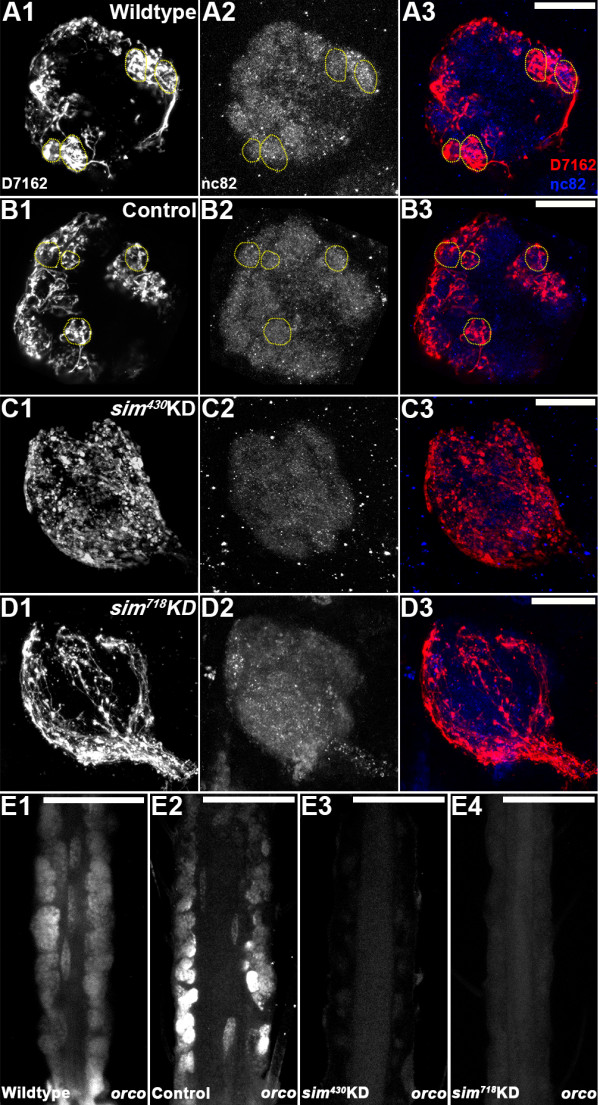
***sim *knockdown pupal antennal lobe phenotypes.** In 36 hr APF wildtype **(A1-A3)** and control-fed **(B1-B3)** pupae, ORNs have innervated the antennal lobe and targeted specific glomeruli within the lobe (D7162 dye fills in **A1**, **B1**; nc82 staining in **A2**, **B2**; overlays are shown in **A3** and **B3**). Distinct glomerular structures are fully formed by this time point (yellow dotted circles in **A1**, **A2**, **B1**, **B2**). D7162 fills **(C1, D1)** of pupal ORNs and nc82 staining of the synaptic neuropil (**C2**, **D2**; overlays in **C3**, **D3**) in *Aae sim* knockdown pupae (*sim*^*430*^ in **C1**-**C3**, *sim*^*718*^ in **D1**-**D3**) revealed sparser and more disorganized ORNs and a collapse in glomerular structure in the antennal lobe. ORNs in *sim* knockdown animals **(C1, D1)** fail to converge on discrete glomeruli. Dorsal is oriented upward in all panels. Scale bars = 25 microns.

Expression of *sim* is detected in both the pupal brain (Figure 6A) and antenna (Figure 6A1). Given this expression and the extent of olfactory system defects observed in larvae, it seemed likely that olfactory developmental phenotypes might also be detected in *sim* knockdown pupae. We therefore examined if olfactory phenotypes could be detected in *sim* knockdown animals at 36 hrs after pupal formation (APF), a time point that more closely resembles the morphology of the adult olfactory system [16]. It was confirmed that chitosan/siRNA-mediated *sim* knockdown in the olfactory system persists through the pupal stages in *sim*^*430*^ (Figure 6C,C1) and *sim*^*718*^ (Figure 6D,D1) nanoparticle-fed animals (compare to control-fed animals in Figure 6B,B1, which resemble wildtype pupae in Figure 6A,A1). Although the full repertoire of *OR* genes assessed in larvae (Figure 3) was not examined in pupae, a continued lack of *orco, OR 9, 10,* and *16* expression was noted in *sim* knockdown pupae 36 hrs APF (Figure 7A3-D3 and A4-D4, respectively; compare to wildtype and control-fed pupae in Figure 7A1-D1 and 7A2-D2, respectively). Furthermore, anterograde labeling was used to trace neurons from the antennae to the developing antennal lobes in wild-type, control-fed, and *sim* knockdown pupae (Figure 8A1-D1). At 36 hrs APF in wildtype (Figure 8A1) and control-fed pupae (Figure 8B1), ORNs have innervated the antennal lobe of the brain and converged on specific glomeruli within the lobe (Figure 8A2,B2; overlays in A3,B3; [2]). D7162 fills of pupal ORNs and nc82 staining of the synaptic neuropil in *sim*^*430*^ (Figure 8C) and *sim*^*718*^ (Figure 8D) knockdown pupae revealed that ORN projections are sparser and more disorganized (Figure 8C1,D1), while glomerular structure is compromised (Figure 8C2,D2; overlays in C3,D3) in the antennal lobes of *sim* knockdown animals. Combined, these results indicate that Sim is required for *A. aegypti* pupal olfactory system development.

## Discussion

### Sim is required for *OR* expression during *A. aegypti* larval and pupal development

Knowledge of the genetic control of *OR* expression during the larval stages of insect development is extremely limited. Moreover, the genes required for *OR* expression had not been assessed during any stage of the mosquito life cycle. Knockdown of *Aae sim* resulted in decreased expression of multiple *OR* genes during *A. aegypti* development (Figures 3 and 7). Transcripts for a number of the *ORs* assayed could not be detected through whole mount *in situ* hybridization in *sim* knockdown animals (Figures 3 and 7). This was the case for *orco* (Figures 3A,B and 7A3,A4), the obligate co-receptor in the assembly and function of heteromeric OR/Orco complexes, which was recently shown to be required for *A. aegypti* human blood meal host preference and their ability to be repelled by volatile DEET [18]. Consensus binding sites for Sim, a PAS-bHLH transcription factor, were identified in over half the *A. aegypti OR* genes, including *orco*, suggesting that Sim likely functions directly as a cis-regulator of *OR* expression (Figure 1). Of course this will need to be tested more carefully in future experiments, which might include generation and comparison of transgenic reporters in which the Sim consensus binding sites in *OR* enhancers are in intact vs. mutated. Such experiments will become more feasible as transgenic technology in mosquitoes advances.

In some cases, the impact of *sim* knockdown on *OR* transcript levels appears to extend beyond the *sim* expression domain. For example, *ORs 10, 60, 89,* and *100* are expressed in proximal antennal sensory neuron cell bodies located outside the normal *sim* expression domain, yet expression of these *ORs* is not detected throughout the *sim* knockdown antenna (Figures 1E and 3C,F,H,J). These data suggest that Sim may also regulate *A. aegypti OR* expression indirectly and non-cell autonomously in some ORNs. The mechanism by which Sim regulates the expression of these *OR* genes remains to be determined. However, the non-cell autonomous impacts of *sim* knockdown on the levels of these *OR* transcripts is not likely to be a non-specific consequence of *sim* knockdown. Loss of *sim* function does not appear to result in antennal cell death (Figure 2E3,E4). Moreover, the normal expression pattern of *OR 90* (which lacks flanking Sim binding sites) in the *sim* knockdown antenna suggests that these antennal sensory neurons begin to differentiate properly (Figure 3I3,I4). Thus, the *sim* knockdown *OR* expression phenotypes reported do not appear to result from non-specific global or neural differentiation defects. This notion is further supported by acetylated tubulin staining data, which detected normal antennal sensory neuron axon bundles in *sim* knockdown antennae (Figure 2F3,F4).

In the *D. melanogaster* developing CNS, Sim is known to repress gene transcription through the activation of repressive factors [6,19-24]. Analysis of *OR* expression in *Aae sim* knockdown animals did not reveal any cases in which *OR* expression was activated in response to loss of *sim* function. Thus, although Sim may have both direct and indirect roles in the regulation of *OR* expression, *sim* knockdown data suggest that it functions as a positive regulator of *OR* expression in *A. aegypti.*

Loss of *sim* resulted in loss of *OR* expression in both larvae (Figure 3) and pupae (Figure 7). These findings suggest that a least one transcription factor functions to regulate *OR* expression at both the larval and pupal stages of insect development. It remains to be seen if this will be the case for other transcription factors expressed in the *A. aegypti* larval antenna (Figure 1). Moreover, it will be interesting to identify the regulatory mechanisms responsible for the differential *OR* gene expression in larval (aquatic) vs. adult (terrestrial) *A. aegypti* mosquitoes that was noted in a previous study [16]. The results of this investigation suggest that the general mechanisms for regulation of *OR* expression may be generally well conserved between dipteran insects. Transcription factors with consensus binding sites flanking mosquito *OR* genes (Table 1), all of which are expressed in the mosquito larval antenna (Figure 1), also function in the *D. melanogaster* pupal/adult regulatory matrix [6,8,23]. Although the roles of these transcription factors remain to be assessed in *Drosophila* larvae, these observations are interesting given the expansion and rapid diversification of mosquito *OR* genes with respect to *D. melanogaster*[15]. The conservation of transcriptional regulation mechanisms may help to explain why comparative analysis of *Drosophila* species suggests that although the sequences of particular ORs have diverged between species, the odor response spectra and organization of ORNs are well conserved [7].

### Coordinate regulation of OR expression and ORN targeting

Although OR expression does not play an instructive role in ORN targeting, the regulation of *OR* gene expression and ORN targeting are tightly coordinated during olfactory system development [10,11]. Pupal ORNs expressing the same *OR* gene project axons that converge on the same glomerulus, one of several spheroidal modules located in the antennal lobe of the insect brain [10,11]. The genetic mechanisms responsible for the coordination of these two processes are not well understood in pupae. Moreover, although there is evidence that *Drosophila* larval ORNs expressing the same *OR* project to similar areas of the larval brain [14], it is unclear how this process is regulated. Our studies indicate that in the absence of Sim, larval and pupal *OR* expression is disrupted, and ORN projections are sparser and more disorganized (Figures 3, 4 and 7). With respect to the antennal lobe phenotype, it is of course difficult to know if Sim is functioning in the antennal sensory neurons or in the brain, as it is expressed in both tissues (Figures 2A,A1 and 6A,A1). Moreover, the sophisticated tissue/cell-specific knockdown experiments that are routine in *Drosophila,* a more genetically tractable system, are not yet possible in the developing mosquito olfactory system. The development of such technologies would permit us to pursue a more complete understanding of the role of Sim in the regulation of ORN targeting. Still, in light of our present observations, it is interesting to consider that two *Drosophila* cis-regulators of *OR* transcription, Acj6 and Pdm3, also function to regulate ORN targeting [6,8,9,23]. Combined, these results suggest that a single transcription factor can function to regulate both ORN targeting and *OR* expression during insect development. Thus, it is possible that the regulatory matrix for *OR* expression in any given insect neuron also serves as the regulatory matrix for ORN targeting of that neuron. In other words, the transcriptional code that controls insect *OR* gene expression in an ORN also regulates axon targeting in that ORN. It will be interesting to determine if the newly identified regulators of *OR* gene expression uncovered in a recent *D. melanogaster* screen [6] also control ORN targeting in mosquitoes and fruit flies. Such a combined transcriptional regulatory mechanism may underlie the precise coordination of *OR* gene expression and ORN targeting observed in the developing insect olfactory system.

### A critical need to understand the regulation of gene expression in vector mosquitoes

Knowledge of developmental gene regulatory regions has resulted in important advancements for the mosquito research community and vector control. For example, Adelman *et al.*[25] used the regulatory regions of the developmental gene *nanos* to drive sex- and tissue-specific expression of transposase in female germ cells, a key innovation in mosquito transgenic technology. Furthermore, the recent design and release of *A. aegypti* bearing a conditional dominant lethal gene that yields a female-flightless phenotype [26-28] evolved from the identification and use of a tissue and sex specific regulatory element, *AaeAct-4,* which drives gene expression in the indirect flight muscles of female pupae. Unfortunately, we presently lack drivers for temporal and tissue-specific gene expression in mosquitoes. The regulation of gene expression is a core aspect of developmental biology, as differential gene expression is central to cell patterning, differentiation, and most developmental processes. Thus expansion of our efforts to study mosquito development will uncover information about cis-regulatory elements. Such knowledge could be applied to the engineering of drivers that may be used to promote gene expression in specific ORNs. Such tools, which have been developed in genetic model organisms such as *D. melanogaster,* would be a tremendous asset to mosquito researchers studying insect olfaction. Knowledge of enhancers in the developing olfactory system may also inform the design of transgenics that could one day be used in integrated vector management strategies.

### siRNA chitosan/nanoparticle gene targeting in *A. aegypti*

The results of this investigation, in conjunction with our recent functional analysis of *sema1a* in *A. aegypti*[2], have demonstrated that chitosan/siRNA nanoparticle-mediated gene targeting can be used to disrupt olfactory system development in insects. The average *sim* knockdown levels obtained in both the brain and antennae were reasonably high in this study, with average knockdown levels in the antennae exceeding those of the brain. These results, in conjunction with previous experiments [2], demonstrate that the central and sensory nervous systems are not refractory to siRNAs. Moreover, within the population of *sim* knockdown animals, one finds that half of the animals display nearly complete loss of *sim* expression in their brains and antennae (Figures 2C,C1,D,D1 and 5B2,B3,C2,C3, Table 3). Thus, use of chitosan/siRNA nanoparticle gene targeting allows for efficient knockdown and the ability to determine the time point in which knockdown is initiated. Controlling the timing of knockdown initiation is helpful for pursuing analysis of developmental regulatory gene function, particularly given that we have not yet developed technology for pursuing mosaic clonal studies in *A. aegypti,* a routine strategy for characterizing embryonic lethal loss of function mutations in *D. melanogaster.* Likewise, for this reason, TALEN-based strategies for site-directed mutagenesis will unfortunately not permit characterization of the late larval or pupal functions of genes for which loss of function mutations result in early developmental lethality. Moreover, the short length of siRNAs makes it more straightforward to design them to be both gene and species-specific, thereby decreasing the potential for off-site targeting, an advantage that is helpful both at the bench and perhaps even someday in the field if issues such as cost and delivery of chitosan/siRNAs could be addressed. For now, chitosan siRNA nanoparticle gene targeting is emerging as a very useful tool for analysis of late larval and pupal development in mosquitoes. This technique could likely be adapted to study olfactory system development in a wide variety of arthropod species. These additional studies are necessary given the paucity of information concerning development of this tissue in most insect species --which is extremely unfortunate given the wealth of diversity that exists in this insect sensory system.

## Conclusions

Chitosan/siRNA-mediated knockdown experiments demonstrated that Sim regulates both larval and pupal olfactory system development in the disease vector mosquito *A. aegypti.* Sim positively regulates the expression of a large subset of larval *OR* genes. The detection of Sim consensus binding sites in the 5’ flanking regions of these *OR* genes suggests that Sim directly activates *OR* gene expression. However, analysis of the expression pattern of Sim suggests that it may also function non-cell autonomously as a regulator of *OR* expression. Thus, Sim may regulate *OR* gene expression through direct and/or indirect mechanisms, a question for future studies. siRNA-mediated *sim* knockdown experiments also revealed antennal lobe defects, including decreased ORN innervation of the larval antennal lobe. These antennal lobe and *OR* expression defects correlated with a larval odorant tracking behavioral defect. *OR* expression and antennal lobe defects were also observed in *sim* knockdown pupae. These results suggest that Sim functions in multiple aspects of *A. aegypti* olfactory system development during both the larval and pupal stages of development.

## Methods

### Mosquito rearing

The *Aedes aegypti* Liverpool-IB12 (LVP-IB12) strain was used in these investigations. Mosquitoes were reared as previously described [29] except that an artificial membrane blood-feeding system was employed in lieu of using vertebrate animals directly.

### Sequence analyses

The 1 kB 5’ flanking sequences immediately upstream of the ORFs of the 115 annotated *A. aegypti OR* genes [15] were exported from VectorBase. These sequences were searched for known transcription factor binding site consensus motifs, which are listed in Table 1. Sites with 100% identity to the consensus motifs were recorded as hits.

### siRNA-nanoparticle-mediated knockdown

Knockdown of *sim* was achieved via chitosan/siRNA-nanoparticle feedings which were performed using the procedure described by Mysore *et al*. [2], which was adapted from Zhang *et al*. [30]. The following siRNAs corresponding to *Aae sim* were synthesized by Dharmacon RNAi Technologies (Lafayette, CO, USA): siRNA *sim*^*430*^ sense: CAACCGAACAUGUUUGCAAUU and antisense: UUGUUGGCUUGUACAAACGUU (corresponds to base pairs 430-448 of *Aae sim*) and siRNA *sim*^*718*^ sense: GGGCACAGUUGCAUCCAAAUU and antisense: UUCCCGUGUCAACGUAGGUUU (corresponds to base pairs 718-736 of *Aae sim*). Control siRNA with no known targets in the *A. aegypti* genome was described previously [31] and used in these experiments. Chitosan/siRNA nanoparticle pellets containing control or knockdown siRNAs were prepared according to the recipe of Zhang *et al.*[30]*. A. aegypti* larvae were fed with control or *sim* knockdown chitosan/siRNA nanoparticles for four hr time periods daily for three days (1 pellet/feeding/50 larvae). For all phenotypes assessed, a minimum of two replicate experiments were performed for both knockdown siRNAs *sim*^*430*^ and *sim*^*718*^. Knockdown was confirmed through whole-mount *in situ* hybridization (n numbers are included in Table 3). Knockdown levels were quantified through qRT-PCR for *sim*^*430*^ nanoparticle-fed animals as described previously [32]. All phenotypes reported in this investigation (n numbers corresponding to each analysis are provided below) were confirmed following treatment with each different knockdown siRNA, and every phenotype reported was observed in over half of the knockdown animals examined (representative pictures are shown), with none of the phenotypes described being observed in wildtype or control embryos.

### Staining and imaging

#### Immunohistochemistry

Immunohistochemical staining experiments were performed as described [16,33]. mAb nc82 (1:50; Developmental Studies Hybridoma Bank, Iowa City, IA, USA) was used for visualization of the synaptic neuropil. Rat anti-5HT (1:100; Abcam, Cambridge, MA, USA) staining marked the serotonergic projection neurons. Anti-cleaved caspase-3 (Cell Signaling Techology, Danvers, MA, USA) is a marker for cell death. Anti-acetylated tubulin (Zymed, San Francisco, CA) staining marks axons. The following secondary antibodies were used at a concentration of 1:200: goat anti-mouse FITC, goat anti-mouse Cy3, goat anti-rabbit FITC (Jackson Immunoresearch, West Grove, PA, USA), and Alexa Fluor 568 goat anti-rat IgG (Life Technologies, Grand Island, NY, USA). Tissues were imaged with a Zeiss 710 confocal microscope using Zen software, and scanned images were analyzed with FIJI and Adobe Photoshop software.

#### In situ hybridization

Digoxygenin-labeled riboprobes corresponding to *Aae E93 (AAEL004572), Aae zf30C (AAEL004774),* as well as the genes listed in Tables 1 and 2, were synthesized as described by Patel [34]. Whole-mount *in situ* hybridization experiments were performed as previously described [35]. Stained tissue preparations were imaged on a Zeiss Axioimager equipped with a Spot Flex camera. In all experiments, a combined total of ~100 larvae (and ~60 pupae) from at least two replicates were analyzed.

### Anterograde tracing of antennal sensory neurons

Sensory neurons of fourth instar and 36 hr pupal antennae were anterogradely traced following application of dextran tetramethylrhodamine (D7162, Life Technologies, Grand Island, NY, USA) as described [36,37]. A total of minimally 20 animals for each condition (wildtype, control-fed, *sim*^*430*^ knockdown, and *sim*^*718*^ knockdown) were examined in two replicate experiments. Dissected brains from these animals were colabeled for expression of additional markers as previously discussed [16].

### Behavioral assay

As described in Mysore *et al*. [2], individual *A. aegypti* fourth instar larvae fed with either control or *sim* knockdown chitosan/siRNA nanoparticles were tested in behavioral assays performed generally as described by Liu *et al.*[17]. In this assay, a yeast odorant pellet is placed on one side of a petri dish, and individual larvae are placed at the opposite end of the dish. Individuals are scored for touching (score = 1) or failing to touch (score = 0) the yeast during the course of a five minute assay. Data collected from four replicate experiments (n = ~45 animals per replicate for each condition) were compiled for statistical analysis using the Student’s *t*-test. Following the behavioral test, *sim* transcript levels were assessed through *in situ* hybridizaton in control vs. *sim* knockdown individuals that had touched or not touched the yeast.

## Abbreviations

OR: Odorant receptor; ORN: Olfactory receptor neuron; Sim: Single-minded; APF: After pupal formation; LVP-IB12: Liverpool-IB12; KD: Knockdown; TF: Transcription factor; ORF: Open reading frame.

## Competing interests

The authors declare that they have no competing interests.

## Authors’ contributions

KM developed the concepts and approach, performed sequence analysis, knockdown, immunohistochemical, targeting, and behavioral experiments, analyzed the data, and prepared the manuscript. EM optimized and performed *in situ* hybridization experiments. PL designed and performed qRT-PCR assays and prepared riboprobes. MDS developed the concepts and approach, performed data analysis, and prepared the manuscript. All authors read and approved the final manuscript.
